# Perri Spanish Auditory Verbal Learning and Memory Test: Normative Data for Elderly Adults from Mexico

**DOI:** 10.3390/healthcare14050583

**Published:** 2026-02-26

**Authors:** Teresita J. Villaseñor-Cabrera, Miguel Ángel Macías-Islas, Karen Sanchez-Jacuinde, Genoveva Rizo-Curiel, Miriam E. Jiménez-Maldonado, Enrique López, Fabiola Gonzalez-Ponce, Jorge I. Gámez-Nava, Laura González-López, Cesar Arturo Nava-Valdivia, Mario A. Mireles-Ramírez, Nayeli Sanchez-Rosales, Jazmin Marquez-Pedroza, Martha Rocio Hernández-Preciado, Edgar Ricardo Valdivia-Tangarife

**Affiliations:** 1 Departamento de Neurociencias, Centro Universitario de Ciencias de la Salud, Universidad de Guadalajara, Guadalajara 44340, Jalisco, Mexico; teresita.villasenor@academicos.udg.mx (T.J.V.-C.); drmaciasbrain@gmail.com (M.Á.M.-I.); karen.sanchez9839@alumnos.udg.mx (K.S.-J.); jimenezme@yahoo.com.mx (M.E.J.-M.); 2 O.P.D Hospital Civil Fray Antonio Alcalde, Guadalajara 44340, Jalisco, Mexico; 3 Departamento de Salud Pública, Centro Universitario de Ciencias de la Salud, Universidad de Guadalajara, Guadalajara 44340, Jalisco, Mexico; genoveva.rizo@academicos.udg.mx; 4 Departamento de Psiquiatria y Neurociencias de la Conducta, Cedars-Sinai Medical Center, Los Angeles, CA 90048, USA; enriquelopez@hotmail.com; 5 Instituto de Terapéutica Experimental y Clínica, Departamento de Fisiología, Centro Universitario de Ciencias de la Salud, Universidad de Guadalajara, Guadalajara 44340, Jalisco, Mexico; fabiola.gonzalez@academicos.udg.mx (F.G.-P.); lauraacademicoudg@gmail.com (L.G.-L.); 6 Programa de Doctorado en Farmacología, Centro Universitario de Ciencias de la Salud, Universidad de Guadalajara, Guadalajara 44340, Jalisco, Mexico; 7 Departamento de Microbiología y Patología, Centro Universitario de Ciencias de la Salud, Universidad de Guadalajara, Guadalajara 44340, Jalisco, Mexico; cesar.navavaldi@academicos.udg.mx; 8 Departamento de Neurología, Unidad de Alta Especialidad Médica, Centro Médico Nacional de Occidente, Instituto Mexicano del Seguro Social, Guadalajara 44340, Jalisco, Mexico; drmmireles@gmail.com (M.A.M.-R.); naye_ale@hotmail.com (N.S.-R.); 9 División de Neurociencias, Centro de Investigación Biomédica de Occidente, Instituto Mexicano del Seguro Social, Guadalajara 44340, Jalisco, Mexico; jaz180688@gmail.com; 10 Departamento de Ciencias Filosóficas, Metodológicas e Instrumentales, Centro Universitario de Ciencias de la Salud, Universidad de Guadalajara, Guadalajara 44340, Jalisco, Mexico; mrociohp@hotmail.com

**Keywords:** elderly, normative, Perri auditory verbal learning test, learning test, verbal memory

## Abstract

**Background:** The Perri Auditory Verbal Learning Test (Perri-AVLT) is a cognitive tool designed to assess verbal learning and memory. Currently, demographically adjusted norms for the Perri-AVLT are not available for elderly Mexican adults. **Objective:** This study aimed to develop regression-based norms from elderly Mexican adults to enable demographic adjustments for clinical interpretation. **Methods:** The sample included 294 elderly Mexican adults aged 60–89 (224 cognitively normal individuals, and 70 clinical cases) from Mexico (Jalisco, Guanajuato, and Mexico City). Participants were administered the Perri-AVLT. A multivariate regression-based norming approach was used to evaluate the effects of age, sex, and years of education on test performance. **Results:** The multivariate regression model showed that years of education were a significant predictor of cognitive performance across all Perri-AVLT trials. The Pearson correlation for all Perri-AVLT trials was high. **Conclusion:** This study provides regression-based normative data for the Perri-AVLT adjusted for sociodemographic factors. These norms can be used to evaluate verbal learning and memory in elderly Mexican adults. This information can support a neuropsychologist in cognitive assessment, rehabilitation, and research.

## 1. Introduction

The Perri Auditory Verbal Learning Test (Perri-AVLT) is a neuropsychological instrument developed to assess verbal learning and memory [1]. The Perri-AVLT provides an assessment of fundamental memory processes, including acquisition, interference effects, retention, and retrieval [2]. Neuropsychological assessment is a crucial component in the detection and diagnosis of neurological conditions [3]. In clinical practice, available neuropsychological instruments must be standardized and validated [4]. The lack of standardized and validated tools for the Spanish-speaking population of Latin America (LATAM) is a significant issue for neuropsychology professionals [5].

Several studies have standardized and validated instruments for assessing verbal memory across different populations [6,7,8,9,10,11,12,13,14,15,16,17]. Nonetheless, only two studies have generated normative data for verbal memory tests in Mexico that specifically included elderly participants. First, Ostrosky-Solis et al. developed, standardized, and analyzed the factor structure of a neuropsychological test battery, named NEUROPSI ATTENTION AND MEMORY. The study included 521 monolingual Spanish-speaking subjects, aged 6 to 85 years. Its verbal memory test consists of three trials of 12 words with high-frequency semantic categories in Spanish (animals, fruits, and body parts), as well as delayed memory, semantic cued recall, and recognition list [18]. Second, Arango-Lasprilla et al. generated normative data on the Hopkins Verbal Learning Test-Revised (HVLT-R) from 11 Latin American countries, including Mexico. The study included 3977 participants, aged 18 to 95 years. The HVLT-R consists of three trials of 12 semantically related words in three categories (profession, sports, and vegetables), followed by a delayed memory test (20–25 min) and a recognition list [19].

Importantly, several structural differences distinguish the Perri-AVLT from other verbal memory tests with available normative data in Mexico. For instance: (a) interference list, the Perri-AVLT includes an interference list (list B), while verbal memory tests of NEUROPSY and HVLT-R do not. Some studies reported that assessing the interference list in verbal memory tests allows evaluation of learning and consolidation capacity and of memory resilience against competing information, thereby enhancing diagnostic sensitivity [20,21]. (b) Immediate and delayed recall by category: the Perri-AVLT includes both immediate and delayed recall by semantic category. In contrast, the NEUROPSY verbal memory test includes only delayed recall by category, whereas the HVLT-R does not include immediate and delayed memory categories. To mitigate executive demands during recall, some verbal memory tests assess recall to semantic category cues, which are thought to reduce cognitive effort by focusing and guiding retrieval [22,23,24].

In 2025, we developed regression-based normative data for the Perri Spanish Auditory Verbal Learning and Memory test in early- to mid-life Mexican adults. We included 430 participants (380 cognitively normal individuals and 50 clinical cases) aged 18–59 years. The results showed that age and years of education were significant predictors of verbal memory performance [17]. However, normative data derived from a younger population cannot be directly extrapolated to older adults, given the well-documented cognitive changes associated with aging. In the context of the progressive aging of the global population, which has led to a substantial increase in the prevalence of age-related cognitive disorders, the absence of age-specific normative data may lead to misclassification, either underestimating subtle cognitive decline or overpathologizing normal aging processes. Therefore, there is a critical need to establish demographically adjusted norms specifically for elderly Mexican adults. The objective of this study was to develop regression-based norms for the Perri AVLT in elderly Mexican adults to enable demographic adjustments and clinical interpretation.

## 2. Materials and Methods

### 2.1. Participants

The present study included 301 elderly Mexican adults aged 60–89; 7 participants were excluded from the analysis due to incomplete sociodemographic information. Resulting in a final sample of 294 elderly Mexican adults, divided into two groups. The first group consisted of 224 cognitively normal, healthy participants stratified by sex and years of education into the following categories: female with fewer than 12 years of education (*n* = 47; 21.0%); female with 12 or more years of education (*n* = 60; 26.8%); male with fewer than 12 years of education (*n* = 53; 23.7%); and male with 12 or more years of education (*n* = 64; 28.6%). Stratification was verified by the absence of a statistically significant association between the demographic variables (X^2^ = 0.043; *p* = 0.836). These participants were recruited from Mexico: Jalisco, Guanajuato, and Mexico City. Cognitively normal, healthy participants were contacted by research staff to determine their interest in participating.

The inclusion criteria for the cognitively normal group were as follows: (a) being born and currently residing in Mexico; (b) speaking Spanish as their native language; (c) being between 60 and 89 years old at the time of the cognitive evaluation; (d) having completed at least one year of formal education; (e) being able to read and write; (f) scoring ≥ 25 on the Mini-Mental State Examination (MMSE) [25,26]; (g) scoring ≥ 90 on the Barthel Index [27,28]; (h) scoring ≤ 4 on the Patient Health Questionnaire-9 (Patient Health Questionnaire-9-PHQ-9) [29,30]; and (i) scoring ≤ 10 on the Generalized Anxiety Disorders-7 (GAD-7) [31,32].

Exclusion criteria for this group were as follows: (a) a history of neurological disorders, developmental or learning disabilities, psychiatric disorders, or chronic medical condition that might affect cognition (e.g., metabolic syndrome, chronic heart failure, or apnea); (b) a history of psychotropic medication use that could impair cognition; (c) substance abuse or dependence; or (d) visual, auditory, or sensory problems.

The second group consisted of 70 Mexican adults diagnosed with Mild Cognitive Impairment (MCI). All MCI patients were evaluated by the Center of Health Sciences (CUCS) in Guadalajara, Mexico. The inclusion criteria for the MCI patients were as follows: (a) a confirmed diagnosis of MCI according to the Petersen’s criteria [33]; (b) an age range 60–89 years at the time of cognitive evaluation; (c) signed informed consent; (d) an MMSE score between 24 and 30; (e) a global cognitive status of 3 on the Global Deterioration Scale (GDS) [34,35]; and (f) impaired cognitive performance (defined as <1.5 SD) in at least one cognitive domain.

Exclusion criteria for this group were as follows: (a) a history of neurological or psychiatric diagnoses; (b) a prior history of intellectual disability; (c) a history of learning problems; (d) a history of abuse of alcohol or other psychotropic substance; (e) have severe behavioral deficits; (f) severe sensory deficits; (g) a history of taking medication for chronic pain.

### 2.2. Procedure

The present study was approved by the Ethics Committee at the university’s Center of Health Sciences (CUCS), University of Guadalajara (approval code CI-04524). This research was designed in accordance with the principles of the 64th Declaration of Helsinki (Last revision Fortaleza, Brazil 2013). All participants (cognitively normal and patients with MCI) who met the inclusion/exclusion criteria underwent cognitive assessment within a standardized time window between 9:00 a.m. and 3:00 p.m.

Data collection from cognitively normal participants began in 2023 and ended in 2024. Data collection from MCI patients began in January 2024 and ended in November 2024. All participants in both groups were volunteers and received no financial compensation for their participation. All participants provided informed consent.

### 2.3. Instruments

The Perri-AVLT assesses learning and memory through a structured series of trials. The test begins with the examiner reading a list of 16 words (List A) drawn from four semantic categories. Participants complete five trials, during which they listen to the list and recall as many words as possible in any order. After these five consecutive trials, a new list of words (List B; the interference list) is presented, and participants attempt to recall as many words as possible from this new list. Immediately following this, participants are prompted to recall only words from List A (Trial 6; immediate free recall). Next, they are asked to recall the words from List A grouped by category (Trial 7; immediate memory category). After a 20-min delay, during which other neuropsychological tests involving non-verbal stimuli are conducted, participants are again prompted to recall words from List A (Trial 8; delayed memory), followed by recalling words from List A by category (Trial 9; delayed memory category). Finally, participants are presented with a list of 40 words, including the 16 words from List A, 8 words from List B, and 16 new words not found in either list. They are then asked to identify the words from List A (“targets”) in a recognition task (Trial 10; delayed recognition).

### 2.4. Statistical Analyses

Statistical analyses were conducted using SPSS version 29 and R version 4.0.3. The assumptions of regression were evaluated using the residuals from each model, not the raw scores. Specifically, the Kolmogorov–Smirnov test was used to assess the normality of the residuals, and Levene’s test was used to evaluate homoscedasticity. In addition, residual plots were visually inspected to confirm linearity and homoscedasticity across the range of predictors. Independence of residuals was tested using the Durbin–Watson statistic, and multicollinearity among predictors was assessed through tolerance values and variance inflation factors. All diagnostics indicated that regression assumptions were adequately met.

Pearson’s correlation for the Perri-AVLT measures was classified as follows: Zero (r = 0–0); Weak (r = 0.10–0.39); Moderate (r = 0.40–0.69); Strong (0.70–0.89); and perfect (r = 0.90–1.0) [36]. Cohen’s d effect sizes were estimated, with values between 0.2 and 0.5 considered to show a small effect, values between 0.5 and 0.8 a medium effect, and values of 0.8 or greater a large effect.

### 2.5. Regression Norming Procedures

The development of regression-based norms followed the methodology described by Van der Elst et al. [37]. Using data from cognitively intact participants (*n* = 224), regression-based norms were established based on their raw scores on the Perri-AVLT. A multivariate regression model was generated to predict raw scores using age, sex (coded 1 = male, 2 = female), and years of education as predictors. To reduce potential multicollinearity and improve the interpretability of the coefficients, age and education were mean-centered. Quadratic terms (age^2^, education^2^) and interaction effects (e.g., age × education, age × sex) were initially considered in the preliminary model. Predictors were retained if they significantly improved model fit, as assessed by likelihood ratio tests (α = 0.01).

The final model yielded unstandardized β weights for each predictor, which were then used—together with the standard deviation of the residuals—to calculate demographically adjusted Z scores for each participant. These Z scores were subsequently converted to T scores (M = 50, SD = 10) to facilitate clinical interpretation.

## 3. Results

### 3.1. Perri AVLT Data

Table 1 presents the mean, standard deviation, and range of Perri-AVLT raw scores for cognitively intact, healthy participants (*n* = 224), stratified by age and years of education. To complement these raw scores, derived measures are frequently computed to provide a more comprehensive evaluation of learning and memory performance [2]. Four key derived scores were calculated: (1) Total Learning (TL), representing the sum of words recalled across trial 1 to 5; (2) Learning Over Trials (LOT), calculated as the different between the TL score and the recall score for Trial 1 (TL − (5 × Trial 1 score); (3) Short-Term Percent Retention (STPR), which reflects Trial 6 recall as a proportion of Trial 5 recall; expressed as a percentage (STPR = 100 × [Trial 6 recall/Trial 5 recall]). (4) Long-Term Percent Retention (LTPR), calculated as the delayed recall memory score (Trial 8) expressed as a proportion of Trial 5 recall (LTPR = 100 × [Trial 8 delayed recall memory/Trial 5 recall]). Descriptive statistics for these summary scores, including both normative and clinical samples, appear in Table 2.

### 3.2. Calculating Normative Performance Using Regression-Based Norms

Three steps are required for calculating the normative performance: (1) Estimating the Predicted Performance: The predicted performance (Scale Score Predicted, (SS_predicted_) was calculated using the regression equation: SS_predicted_ = Intercept + (individual sex × sex coefficient) + (age centered × age coefficient) + (years of education centered × education coefficient) + (coefficient for Trial n) + (years of education × coefficient for education for Trial) + (individual sex × sex coefficient for Trial n), (2) Subtracting the Observed Score from the Predicted Score: The observed scale score (Scale Score Actual, SS_actual_) was subtracted from the predicted scale score (SSpredicted), and (3) Standardization: Z-scores were calculated using formula: Z = SS_actual_ − SS_predicted_/standard deviation of residual. These Z-scores were subsequently converted to T-scores with a mean of 50 and standard deviation of 10 using the formula: T = Z × 10 + 50.

In the analyses, age and years of education were mean-centered using the following values: mean age (M = 64.71 ± 5.41 years); mean years of education (M = 11.52 ± 5.45 years). These centered values were used to calculate all regression coefficients. The regression coefficient from the multiple regression model was applied using the formula: SS_predicted_ = Intercept (individual sex × sex coefficient) (age centered × age coefficient) (years of education centered × education coefficient) (coefficient for Trial n) (years of education × coefficient for education for Trial) (individual sex × sex coefficient for Trial n).

As an example, consider a 65-year-old female with 12 years of education who recalled 10 words on Trial 2. To calculate the predicted performance and T-score, the following steps were taken:

(1) Centering Variables: The participant’s age was centered as follows: Age centered = 65 − 64.71 = 0.29. Similarly, years of education were centered as follows: Year of education centered = 12 − 11.52= 0.48.
(2) Predicted Score (SSpredicted): Using the regression formula, the predicted score (SSpredicted) was calculated: SS_predicted_ = 8.653 + (1 × 0.677) + (0.29 × −0.013) + (0.48 × 0.148) + 3.428 + (0.48 × 0.326) + (1 × 0.0.716) = 13.60] (Table 3 and Table 4).
(3) Standardized Residual (Z-score): The actual observed score (SSactual) was subtracted from the predicted score and divided by the standard deviation of residual for Trial 2: Z = SSactual -SSpredicted/standard deviation of residual.

Substituting values: Z = (10 − 13.60)/2.39015) = −1.5061

Converting to T-score: The Z-score was then converted to a T-score using the following formula: T = (Z*10) + 50. Substituting values: T = (−1.5438*10) + 50 = 34.93

Thus, the participant’s T-score for Trail 2 was 34.93, reflecting their performance adjusted for age, sex, and years of education.

### 3.3. Estimate Test–Retest Reliability and Practice Effect

A sub-sample of cognitively intact, healthy participants (*n* = 60) completed a follow-up assessment of the Perri-AVLT within a two-week interval, allowing for test–retest reliability analyses. This test–retest sample consisted of 34 males (68.0%) and 16 females (32.0%) with a mean age of 65.22 years (SD = 6.36) and a mean of 13.14 years of education (SD = 2.58). Reliability coefficients for the primary Perri-AVLT measures were calculated across the two testing sessions. Performance metrics at Time 1 and Time 2, along with their correlation coefficients (Table 5).

### 3.4. Clinical Sample

Student’s test showed statistically poor performance by the clinical sample compared to cognitively intact participants. This significant difference was observed on the following trials: trial 1 (*p* = 0.036); trial 2 (*p* < 0.001); trial 3 (*p* < 0.001); trial 4 (*p* < 0.001); trial 5 (*p* < 0.001); list B (*p* = 0.002); trial 6 (*p* < 0.001); trial 7 (*p* < 0.001); trial 8 (*p* < 0.001); trial 9 (*p* = 0.018) and trial 10 (*p* = 0.023) (Table 6). The groups did not differ about age (Clinical sample= 64.96 ± 5.70 years; Cognitively intact, healthy participants = 64.54 ± 5.00 years, t = 0.457; *p* = 0.649); years of education (Clinical sample = 9.37 ± 4.16 years; Cognitively intact, healthy participants= 9.49 ± 4.14, t = −0.163; *p* = 0.871); and sex (Clinical sample = 49.5% female; Cognitively intact, healthy participants= 72.9% female, X^2^ = 0.310; *p* = 0.577). The cognitively intact participants included in this comparison represent a subsample of the larger group of cognitively intact participants.

As an example, consider a 65-year-old female with MCI who has 12 years of education and scores a 5 on trial 2. To calculate the predicted performance and T-score, the following steps were taken:

1. Centering Variables: The participant’s age was centered as follows: Age centered = 65 − 64.71 = 0.29. Similarly, years of education were centered as follows: Year of education centered = 12 − 11.52 = 0.48.
2. Predicted Score (SSpredicted): Using the regression formula, the predicted score (SSpredicted) was calculated: SS_predicted_ = 8.653 + (1 × 0.677) + (0.29 × − 0.013) + (0.48 × 0.148) + 3.428 + (0.48 × 0.326) + (1 × 0.0.716) = 13.60].
3. Standardized Residual (Z-score): The actual observed score (SSactual) was subtracted from the predicted score and divided by the standard deviation of residual for Trial 2: Z = SSactual -SSpredicted/standard deviation of residual.

Substituting values: Z = (6 − 13.60)/2.39015) = −3.1797

Converting to T-score: The Z-score was then converted to a T-score using the following formula: T = (Z*10) + 50. Substituting values: T = (−3.1797 × 10) + 50 = 18.20

Thus, the participant’s T-score for Trail 2 was 18.20, reflecting their performance adjusted for age, sex, and years of education.

## 4. Discussion

The world population is rapidly aging. Previous studies have suggested that variation in cognitive performance among older adults may be influenced by childhood conditions, nutrition, the duration and quality of education, exposure to disease, and patterns of physical and social activity [38,39]. In the present study, we developed regression-based norms for the Perri-AVLT in elderly Mexican adults aged 60–89 years. In this context, two previous studies have provided data for the Perri-AVLT in the Mexican population. The first adapted and obtained normative data in Mexican children aged 6–11 years, while the second developed regression-based normative data in Mexican adults aged 18–59 years [17,24]. Consistent with previous research on regression-based normative approaches [40,41,42,43], the present study generated normative data based on a sample of 240 elderly Mexican adults aged 60–89. Some studies have been conducted to provide normative data for elderly Spanish speakers [44,45,46,47].

Our results showed that women outperformed men on trials 1,2, 6, and 7. Consistent with these findings, our group previously reported that women outperformed men on trials 5 and 6 [17]. Several studies have indicated that females demonstrated better performance on memory verbal tasks [41,48,49,50,51]. A study conducted by Boenniger et al. reported that women learned, on average, 1.263 more words than men [52]. In contrast, Perri et al. generated local norms for the Perri-AVLT in a study that included 100 native Spanish-speaking adults from Denver, USA [1]. Their results showed no significant sex differences in any of the Perri AVLT measures. However, the sample sizes differed substantially between studies. The present study included 224 older adults (aged 60–89), whereas Perri et al. analyzed a subsample of only 3 older adults (aged 60–70).

In our study, the total learning and education explained 32.3% of the total variance in scores. In all trials, years of education were an important predictor of cognitive performance. Several studies had reported that education explained a substantial proportion of the variance in scores [13,41,53]. On the other hand, we did not observe a significant age effect on the total learning. Also, Espenes et al. reported a weak effect of age on total learning in a sample of 244 healthy adults aged 49–79 years from Norway and Sweden [8]. However, a study conducted by Welsh et al. included 430 control participants aged 50–89 years. They reported that verbal learning and memory tests were the cognitive domains most affected by age [54]. The absence of a significant age effect on total learning in our study may be attributed to several factors. First, years of education emerged as a strong predictor of performance. We included 124 participants (55.4%) with 12 or more years of education, suggesting a relatively high education level that may reflect greater cognitive reserve. Second, the inclusion of cognitively healthy participants may have minimized the influence of pathological aging processes that typically contribute to age-related declines in verbal memory performance.

With the continuous increase in life expectancy, the proportion of older adults is also increasing. This trend in turn makes the neurodegenerative conditions of old age, thereby increasing the need for standardized neuropsychological tests [55,56]. In this study, we compared the cognitive performance between MCI participants and cognitively intact elderly adults. Our results indicated that MCI participants performed more poorly across all Perri-AVLT trials. A study conducted by Ribeiro et al. reported that MCI participants have learning difficulties and deficient use of semantic clustering strategies [57]. These findings suggest that Perri-AVLT can help identify individuals with MCI and cognitively healthy older adults.

### Limitations and Future Directions

The present study has several limitations that warrant consideration. First, the sample size (*n* = 224) limits the degree to which normative data reflect population-level parameters. Second, participants were recruited exclusively from Jalisco, Guanajuato, and Mexico City, which may not fully represent the broader Mexican population. Third, this study only generated regression-based norms for older cognitively intact adults; thus, the findings cannot be generalized to pediatric populations. Addressing these limitations in future studies can provide a more comprehensive understanding of the Perri-AVLT’s applicability. Fourth, a comprehensive neuropsychological assessment was not conducted to confirm the cognitive status of participants classified as cognitively intact elderly adults. This limitation should be considered when interpreting the normative data, as undetected deficits in some participants could have influenced the results.

## 5. Conclusions

The present study is the first to generate regression-based normative data for the Perri-AVLT using a sample of cognitively intact Mexican adults aged 60–89. The proposed regression models incorporate key demographic predictors (age, years of education, and sex), allowing for more precise demographic adjustments in test interpretation. These norms may support clinical assessment and research application in the older Mexican population. Future studies should explain these norms to include diverse populations and broader age ranges, ensuring the test’s applicability across the lifespan.

## Figures and Tables

**Table 1:** Means (M), Standard Deviations (SD), and Ranges (R) for the normative sample (*n* = 163), stratified by education and age groups.

	Trials					List	Trial	Trial	Trial	Trial	Trial
	1	2	3	4	5	B	6	7	8	9	10
Full Sample Age 60–89 years (*n* = 224)											
M	5.13	6.83	8.17	8.88	9.57	4.65	8.13	8.51	8.00	8.49	14.35
SD	1.79	1.80	1.89	2.05	2.13	1.42	2.51	3.16	2.87	3.41	1.57
R	3–11	4–12	5–13	5–15	6–16	2–9	4–16	4–16	4–15	4–16	10–16
Education < 12 years Age 60–89 years (*n* = 100)											
M	4.61	5.65	7.01	7.56	8.10	4.02	6.07	5.46	5.31	5.21	13.67
SD	1.50	1.00	0.87	1.18	0.88	0.92	1.16	0.93	0.88	0.75	1.71
R	3–8	4–8	5–9	5–10	7–10	2–6	4–8	4–7	4–7	4–7	10–16
Education ≥ 12 years Age 60–89 years (*n* = 124)											
M	5.56	7.79	9.10	9.95	10.76	5.16	9.78	10.97	10.17	11.14	14.90
SD	1.90	1.71	1.97	1.97	2.10	1.54	2.03	1.96	1.92	2.21	1.19
R	3–11	4–12	5–13	5–15	6–16	2–9	6–16	7–16	6–15	6–16	11–16

Note: Trial 1: Number of correctly recall words from list A after first learning trial; Trial 2: Second learning trial; Trial 3: Third learning trial; Trial 4: Fourth learning trial; Trial 5: Fifth learning trial: List B: Free recall of list B; Trial 6 (Immediate memory): Recall of list A without renewed presentation; Trial 7 (Immediate memory category): Recall of list A without renewed presentation by category; Trial 8 (Delayed memory): Recall of list A without renewed presentation after twenty minutes; Trial 9 (Delayed memory category): Recall of list A without renewed presentation after twenty minutes; Trial 10 (Delayed recognition): Recognition of 16 words of list A.

**Table 2:** Summary Scores for Total Learning (TL), Learning Over Trials (LOT), Short-Term Percent Retention (STPR), and Long-Term Percent Retention (LTPR).

	Total Learning	Learning Over Trials	Short-Term Percent Retention	Long-Term Percent Retention
Full Sample Age 60–89 years (*n* = 224)				
M	38.59	12.92	89.90	83.53
SD	7.95	7.56	24.39	24.45
R	25–62	1–32	40–150	44–155
Education < 12 years Age 60–89 years (*n* = 100)				
M	32.93	9.88	76.13	66.46
SD	3.67	4.95	18.18	14.24
R	28–42	1–17	40–114	44–100
Education > 12 years Age 60–89 years (*n* = 124)				
M	43.16	15.37	93.78	97.29
SD	7.53	8.39	22.42	22.20
R	25–62	1–32	46–150	61–155

**Table 3:** Coefficients from multivariate regression for normative adjustments on primary variables of the Perri AVLT, based on data from 224 cognitively normal participants.

Parameter	b	b 95% CI [LL, UL]	s.e.	t	*p*	SD Residual
Intercept	8.563	[3.789, 13.337]	2.422	3.535	<0.001	2.76810
Age	−0.013	[−0.085, 0.056]	0.036	−0.403	0.687	
Education	0.148	[0.081, 0.216]	0.034	4.332	<0.001	
Sex	0.677	[−0.086, 1.441]	0.387	1.748	0.083	
Trial 2	3.428	[2.189, 4.666]	0.628	5.455	<0.001	2.39015
Trial 3	4.764	[3.197, 6.332]	0.795	5.989	<0.001	2.67332
Trial 4	4.891	[3.314, 6.468]	0.800	6.111	<0.001	2.68611
Trial 5	6.708	[4.986, 8.429]	0.874	7.678	<0.001	2.82939
List B	6.775	[5.523, 8.026]	0.635	10.669	<0.001	2.76629
Trial 6	6.980	[5.733, 8.277]	0.633	11.030	<0.001	2.78310
Trial 7	7.157	[6.116, 8.198]	0.528	13.544	<0.001	2.74884
Edu×Trial 2	0.326	[0.269, 0.384]	0.029	14.945	<0.001	
Edu×Trial 3	0.285	[0.227, 0.343]	0.030	9.666	<0.001	
Edu×Trial 4	0.344	[0.289, 0.399]	0.028	12.300	<0.001	
Edu×Trial 5	0.322	[0.266, 0.378]	0.028	11.312	<0.001	
Edu×List B	0.204	[0.140, 0.267]	0.032	6.335	<0.001	
Edu×Trial 6	0.430	[0.381, 0.478]	0.025	17.437	<0.001	
Edu×Trial 7	0.498	[0.455, 0.540]	0.021	23.148	<0.001	
Sex×Trial 2	0.716	[−0.057, 1.490]	0.393	1.824	0.690	
Sex×Trial 3	−0.185	[−0.939, 0.569]	0.383	−0.484	0.629	
Sex×Trial 4	−0.319	[−1.096, 0.458]	0.394	−0.810	0.419	
Sex×Trial 5	−0.557	[−1.321, 0.206]	−0.96	−1.438	0.152	
Sex×List B	−0.224	[−0.973, 0.526]	0.380	−0.588	0.557	
Sex×Trial 6	0.645	[−0.164, 1.455]	0.105	1.571	0.118	
Sex×Trial 7	0.518	[−0.331, 1.368]	0.431	1.202	0.231	

Note: Intercept represents reference category Trial 1; b = unstandardized beta coefficient; s.e. = standard error of the unstandardized beta coefficient; SD residual = standard deviation of the residuals; Trial 1: Number of correctly recall words from list A after first learning trial; Trial 2: Second learning trial; Trial 3: Third learning trial; Trial 4: Fourth learning trial; Trial 5: Fifth learning trial: List B: Free recall of list B; Trial 6 (Immediate memory): Recall of list A without renewed presentation; Trial 7 (Immediate memory category): Recall of list A without renewed presentation by category; Trial 8 (Delayed memory): Recall of list A without renewed presentation after twenty minutes; Trial 9 (Delayed memory category): Recall of list A without renewed presentation after twenty minutes; Trial 10 (Delayed recognition): Recognition of 16 words of list A.

**Table 4:** Coefficients from multiple regression analysis for derived Perri-AVLT measures, based on data from 224 cognitively normal participants.

Parameter	b	b 95% CI [LL, UL]	s.e.	t	*p*	Partial R^2^	Adj. R^2^	SD Residual
TL intercept	0.473	[0.386, 0.559]	0.466	10.733	<0.001		0.365	1.80357
TL age	0.001	[−0.045, 0.047]	0.023	0.057	0.955	−0.004		
TL education	0.283	[0.237, 0.329]	0.023	12.152	<0.001	0.427		
TL sex	−0.258	[−0.760, 0.244]	0.255	−1.012	0.313	0.000		
LOT intercept	−0.676	[−0.763, −0.588]	0.044	−15.209	<0.001		0.578	1.82033
LOT age	0.001	[−0.045, 0.048]	0.024	0.057	0.955	0.003		
LOT education	0.286	[0.239, 0.332]	0.024	12.152	<0.001	0.129		
LOT sex	−0.260	[−1.015, 0.310]	0.257	−1.012	0.313	0.019		
STPR intercept	−0.042	[−0.169, 0.085]	0.065	−0.650	0.517		0.180	2.64799
STPR age	0.039	[−0.029, 0.301]	0.034	1.134	0.258	0.001		
STPR education	0.212	[0.162, 0.297]	0.034	6.704	<0.001	0.169		
STPR sex	0.229	[0.010, 0.241]	0.374	2.616	0.010	0.015		
LTPR intercept	−0.011	[−0.121, 0.100]	0.056	−0.192	0.848		0.403	2.29412
LTPR age	0.023	[−0.036, 0.082]	0.030	0.770	0.442	0.000		
LTPR education	0.347	[0.289, 0.406]	0.030	11.725	<0.001	0.395		
LTPR sex	0.856	[0.217, 1.495]	0.324	2.642	0.009	0.011		

Note: Intercept represents reference category Trial 1; b = unstandardized beta coefficient; s.e. = standard error of the unstandardized beta coefficient; SD residual = standard deviation of the residuals; TL (Total learning) = Trial 1 to 5); LOT (TL-(5 ×Trial 1) = learning over trial; STRP (calculated as percentage of trial 6) = Short-term percent retention; LTPR (calculated as percentage of trial 8) = long-term percent retention.

**Table 5:** Estimate test–retest reliability (*n* = 50).

Perri-AVLT	Time 1	Time 2	r
Trial 1	6.12 ± 1.81	5.92 ± 1.49	0.74
Trial 2	7.56 ± 1.90	6.96 ± 1.60	0.62
Trial 3	8.78 ± 1.92	8.34 ± 1.63	0.70
Trial 4	9.32 ± 1.72	9.24 ± 1.37	0.79
Trial 5	9.86 ± 1.71	9.48 ± 1.55	0.74
List B	4.94 ± 1.73	5.08 ± 2.26	0.77
Trial 6	9.82 ± 1.41	9.58 ± 1.32	0.86
Trial 7	10.78 ± 2.07	10.40 ± 1.94	0.82
Trial 8	9.78 ± 1.54	9.52 ± 1.34	0.80
Trial 9	11.12 ± 2.02	11.26 ± 2.12	0.63
Trial 10	15.04 ± 1.08	15.04 ± 1.14	0.92

Note: Trial 1: Number of correctly recall words from list A after first learning trial; Trial 2: Second learning trial; Trial 3: Third learning trial; Trial 4: Fourth learning trial; Trial 5: Fifth learning trial: List B: Free recall of list B; Trial 6 (Immediate memory): Recall of list A without renewed presentation; Trial 7 (Immediate memory category): Recall of list A without renewed presentation by category; Trial 8 (Delayed memory): Recall of list A without renewed presentation after twenty minutes; Trial 9 (Delayed memory category): Recall of list A without renewed presentation after twenty minutes; Trial 10 (Delayed recognition): Recognition of 16 words of list A.

**Table 6:** Raw scores for the clinical sample and cognitively normal participants.

	Clinical Sample (*n* = 70)	Cognitively Intact, Healthy Participants (n70)	t Student	*p*	Cohens-d
Trial 1, M ± SD	3.87 ± 1.64	4.41 ± 1.38	−2.113	0.036	1.52
Trial 2, M ± SD	4.94 ± 1.50	6.26 ± 1.15	−5.810	<0.001	1.33
Trial 3, M ± SD	6.00 ± 1.81	7.80 ± 1.43	−6.528	<0.001	1.63
Trial 4, M ± SD	6.40 ± 1.87	8.53 ± 1.69	−7.050	<0.001	1.78
Trial 5, M ± SD	6.69 ± 2.25	9.61 ± 2.28	−7.640	<0.001	2.26
List B, M ± SD	3.63 ± 1.97	4.53 ± 1.25	−3.210	0.002	1.65
Trial 6, M ± SD	4.91 ± 2.85	7.01 ± 2.06	−4.987	<0.001	2.49
Trial 7, M ± SD	5.93 ± 2.65	7.51 ± 2.54	−3.610	<0.001	2.59
Trial 8, M ± SD	4.81 ± 3.77	6.94 ± 2.18	−4.084	<0.001	3.08
Trial 9, M ± SD	5.34 ± 4.20	7.11 ± 2.49	−1.321	0.018	3.45
Trial 10, M ± SD	12.27 ± 5.61	13.87 ± 1.58	−2.296	0.023	4.12

Note: Trial 1: Number of correctly recall words from list A after first learning trial; Trial 2: Second learning trial; Trial 3: Third learning trial; Trial 4: Fourth learning trial; Trial 5: Fifth learning trial: List B: Free recall of list B; Trial 6 (Immediate memory): Recall of list A without renewed presentation; Trial 7 (Immediate memory category): Recall of list A without renewed presentation by category; Trial 8 (Delayed memory): Recall of list A without renewed presentation after twenty minutes; Trial 9 (Delayed memory category): Recall of list A without renewed presentation after twenty minutes; Trial 10 (Delayed recognition): Recognition of 16 words of list A.

## Data Availability

Due to ethical restrictions and the sensitive nature of neuropsychological and clinical data, the datasets generated and analyzed during the study are not publicly available. However, the minimal anonymized dataset supporting the main findings of the study is available from the corresponding author upon reasonable request, subject to approval by the local ethics committee and in accordance with institutional and data protection regulations.
